# Correction: Plasticity-Driven Self-Organization under Topological Constraints Accounts for Non-random Features of Cortical Synaptic Wiring

**DOI:** 10.1371/journal.pcbi.1004810

**Published:** 2016-03-04

**Authors:** The PLOS Computational Biology Staff

There is a minor error in the vertical axis label of the lower right portion of Fig 5. Please view the correct version of Fig 5 below:

**Fig 5:**
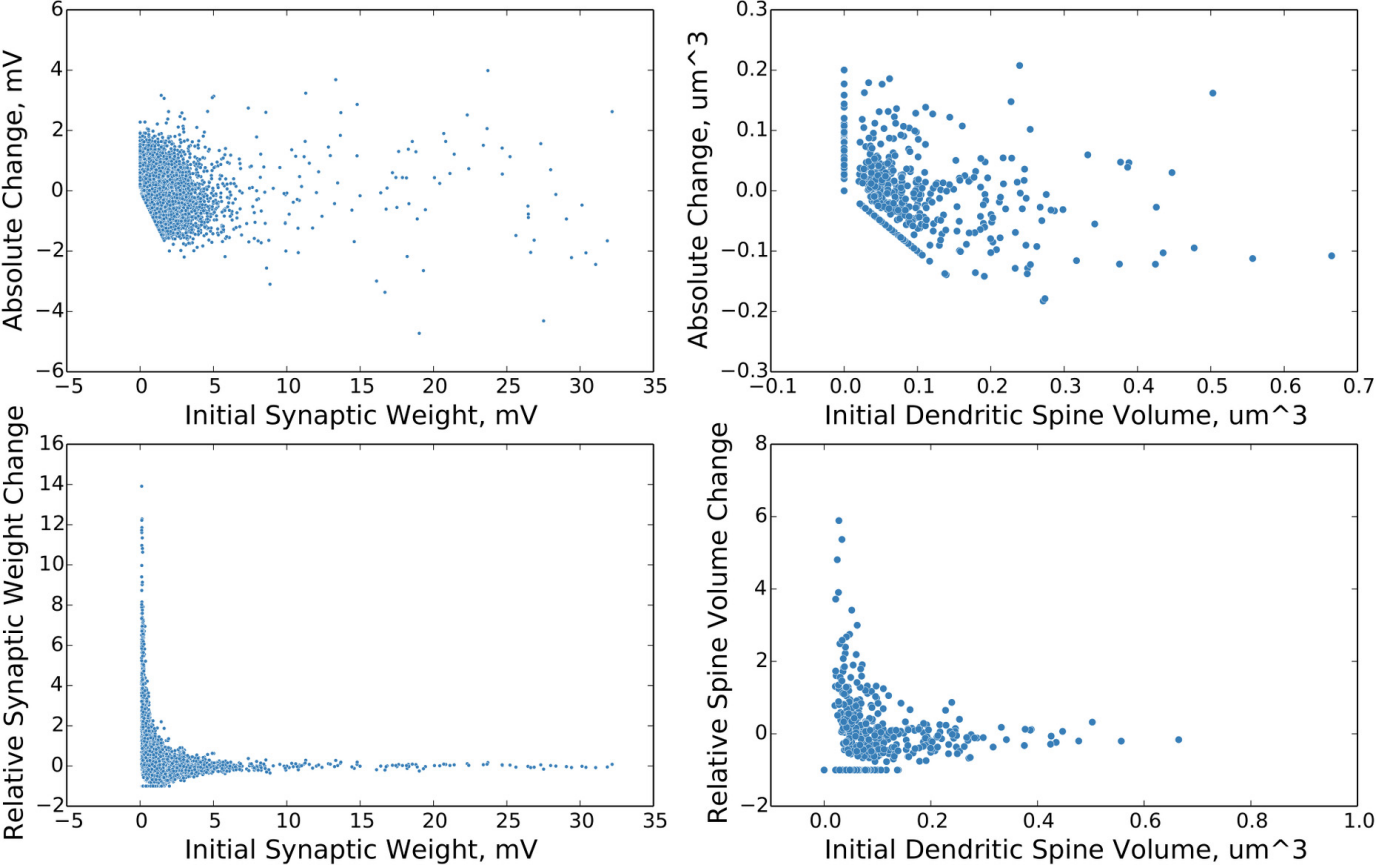
Change in synaptic weight as a function of initial synaptic weight. The above plots show the distributions of change in synaptic weight as a function of initial synaptic weight over a ten second simulation time period. The plots on the left are from the simulated network and are in electrophysiological units. The plots on the right are from experiment [10] and are in units of volume as estimated from fluorescence data. The plots on the top show the absolute change in synaptic weight / size. The plots on the bottom show the relative change in synaptic weight / size. Single trial data.
