# Supplementary figures and images for: Plasticity-Driven Self-Organization under Topological Constraints Accounts for Non-random Features of Cortical Synaptic Wiring
Source: PLoS Comput Biol. 2016 Feb 11;12(2):e1004759. doi: 10.1371/journal.pcbi.1004759 (PMC4750861; doi:10.1371/journal.pcbi.1004759)

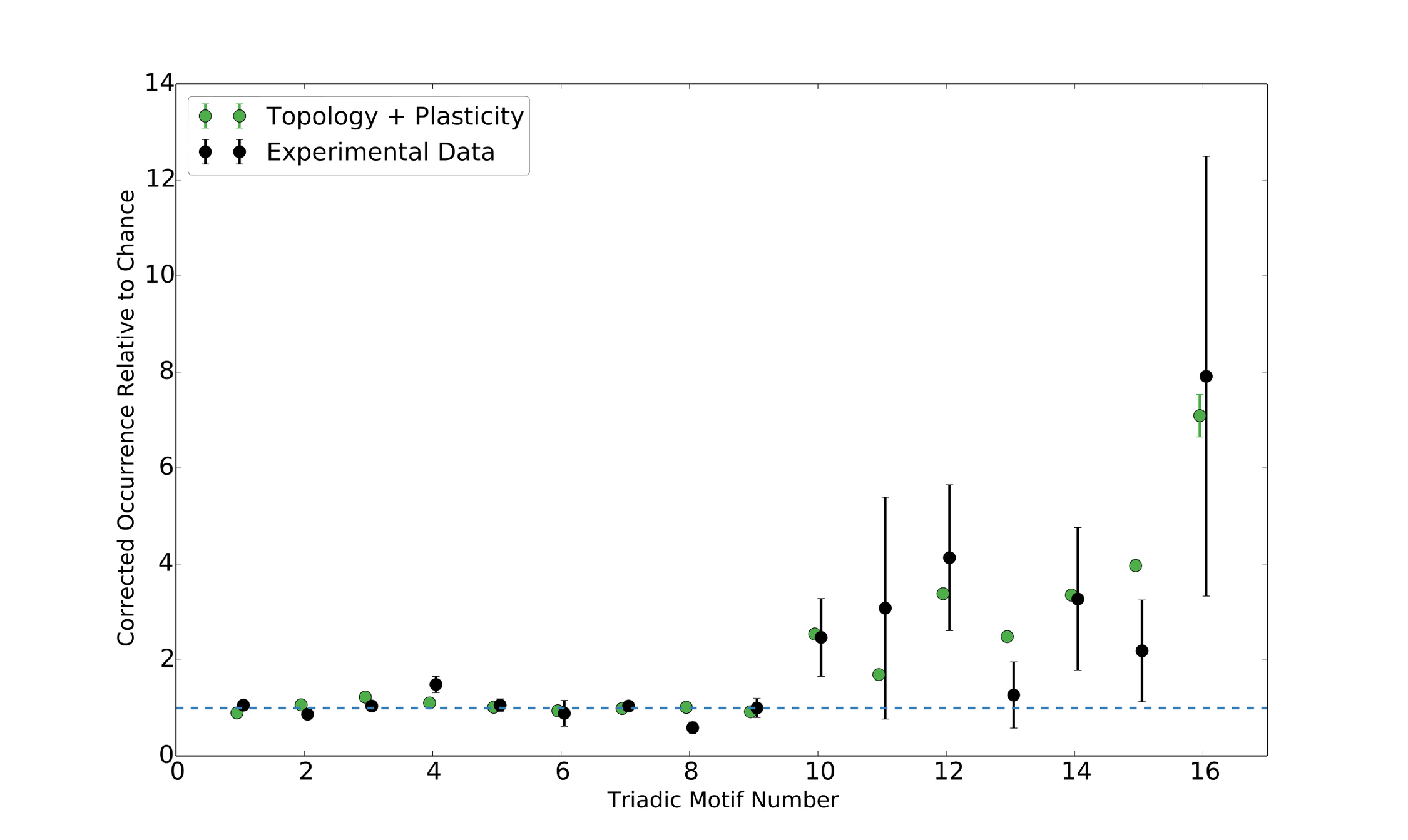

Supplement: S1 Fig — Triadic motif counts (in the same order as [6]) for a simulated network as a multiple of chance value. The counts have been corrected for the observed overrepresentation of bidirectional connections. Results are shown for a complete network with IP target rates drawn from a log-normal distribution (mean of 3.0, standard deviation of 1.0 Hz) instead of a single value and approximate experimental data. Other parameters remain the same, aside from scaling of growth rate to obtain stable phase connection fraction of 0.1. Error bars are standard deviation. Horizontal axis has been jittered slightly to increase readability. (TIF) [file pcbi.1004759.s001.tif]

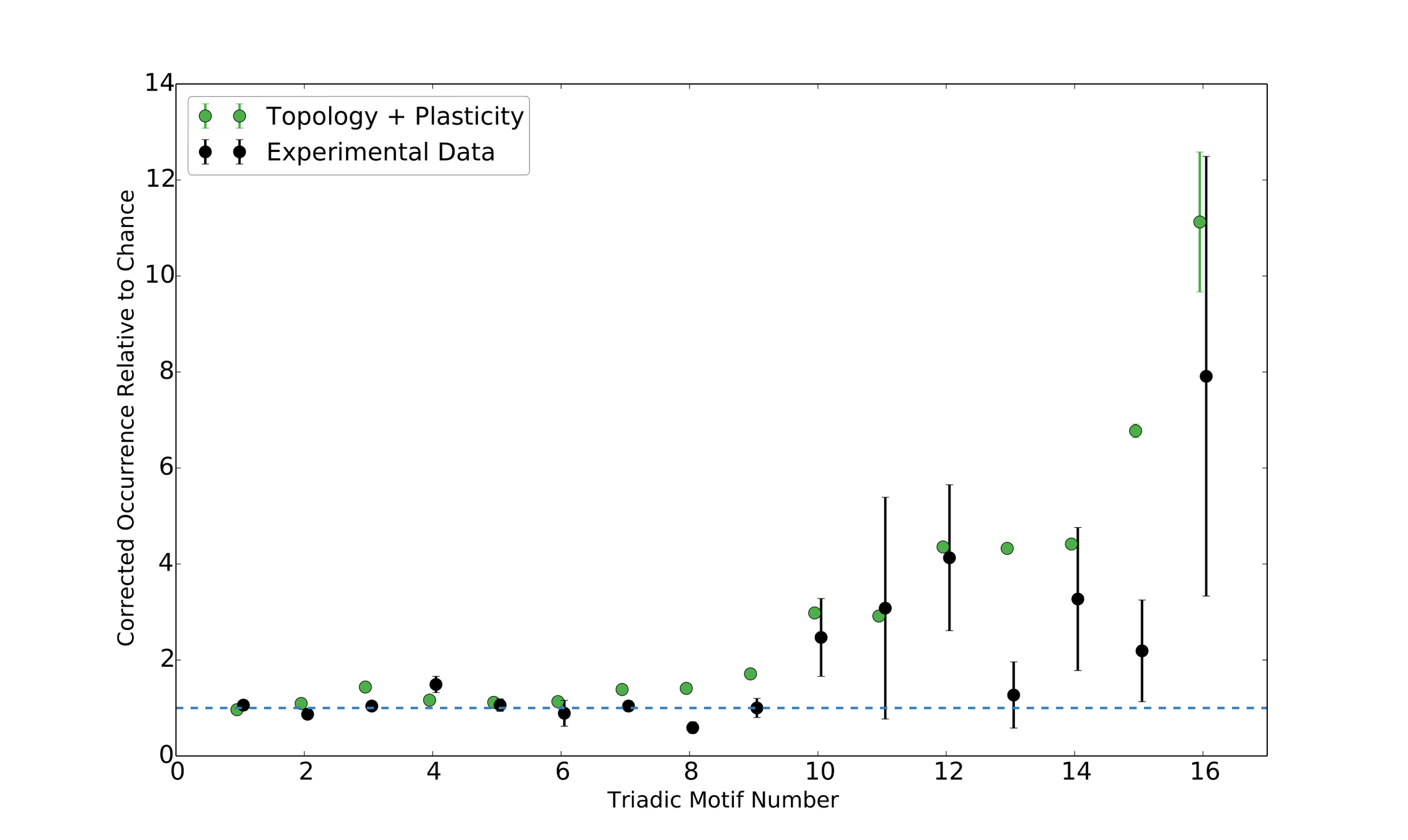

Supplement: S2 Fig — Triadic motif counts (in the same order as [6]) for a simulated network as a multiple of chance value. The counts have been corrected for the observed overrepresentation of bidirectional connections. Results are shown for a complete network of 2000 neurons and approximate experimental data. Other parameters remain the same, aside from scaling of growth rate to obtain stable phase connection fraction of 0.1. Error bars are standard deviation. Horizontal axis has been jittered slightly to increase readability. (TIF) [file pcbi.1004759.s002.tif]

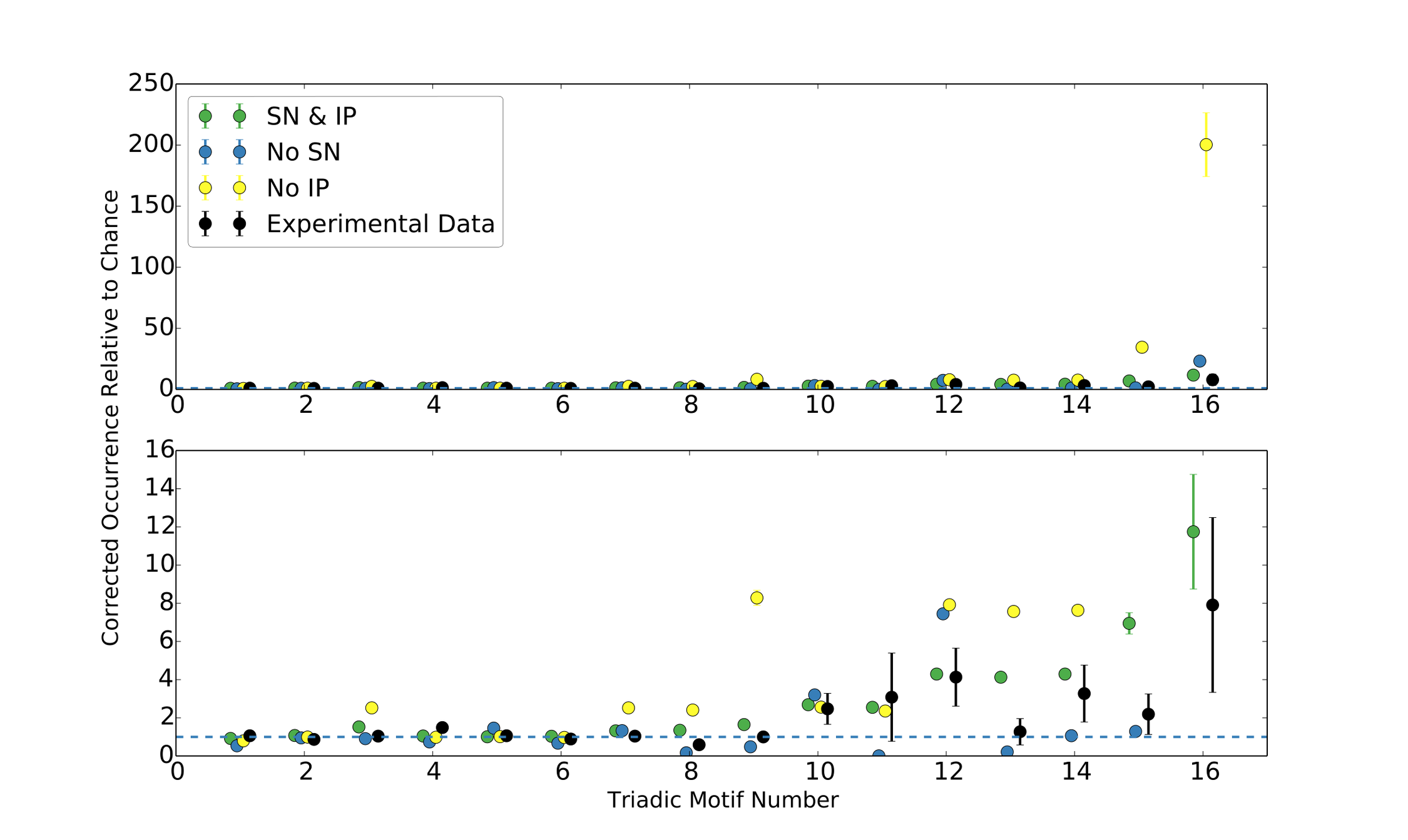

Supplement: S3 Fig — Triadic motif counts (in the same order as [6]) for a simulated network as a multiple of chance value. The counts have been corrected for the observed overrepresentation of bidirectional connections. Results are shown for a network with all plasticity mechanisms, a network without IP, a network without SN, and approximate experimental data. Error bars are standard deviation. Horizontal axis has been jittered slightly to increase readability. Upper and lower plot show the same data with a different scaling of the y-axis. (TIF) [file pcbi.1004759.s003.tif]

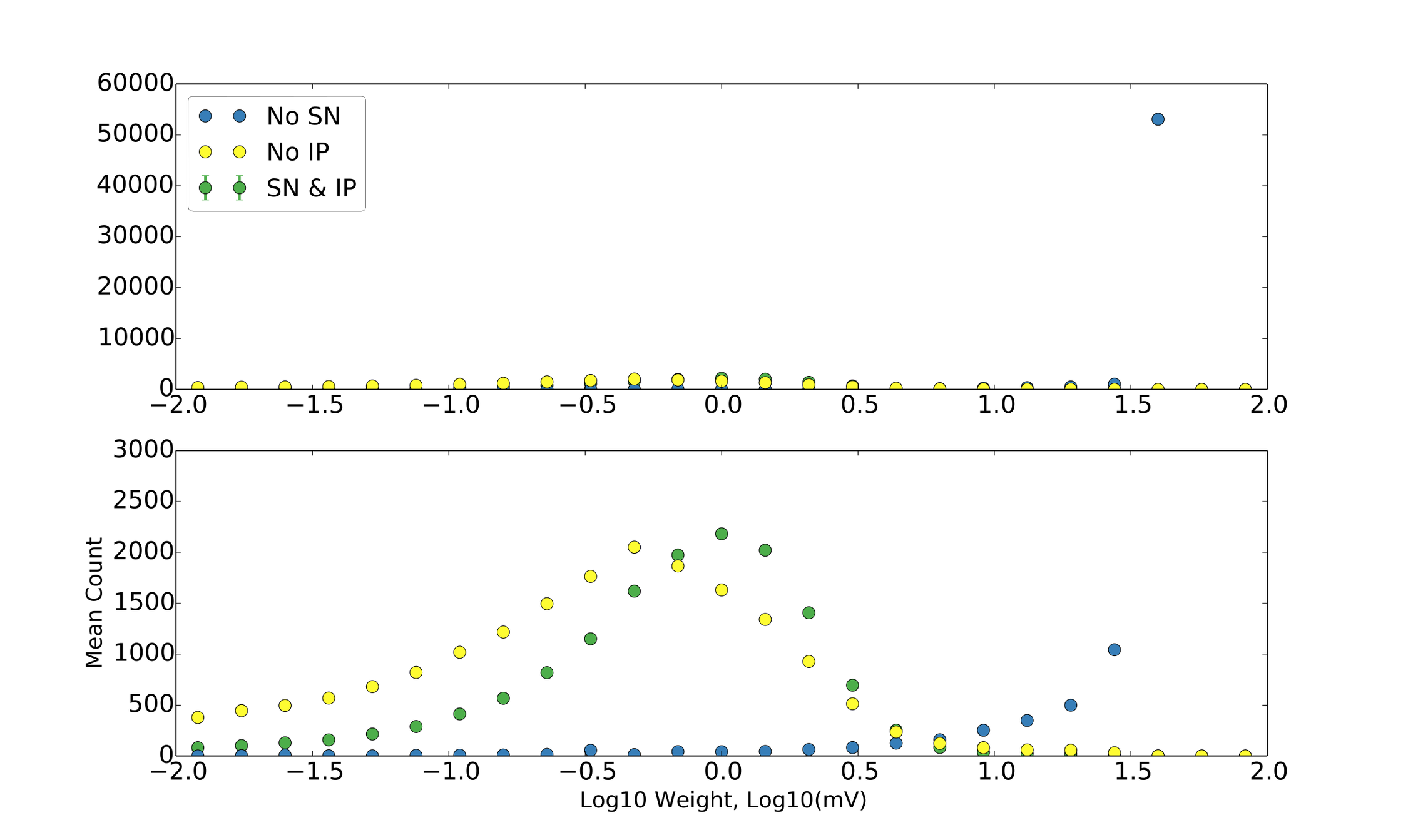

Supplement: S4 Fig — The distribution of the base ten logarithm of synaptic weights for a network all plasticity mechanisms (ten trials), a single network without IP, and a single network without SN. Error bars are standard deviation. Upper and lower plot show the same data with a different scaling of the y-axis. (TIF) [file pcbi.1004759.s004.tif]
