# Supplementary material for: Plasticity-Driven Self-Organization under Topological Constraints Accounts for Non-random Features of Cortical Synaptic Wiring
Source: PLoS Comput Biol. 2016 Feb 11;12(2):e1004759. doi: 10.1371/journal.pcbi.1004759 (PMC4750861; doi:10.1371/journal.pcbi.1004759)
Supplement: S1 Table — Representative single trial example data. Spike correlation was taken from 50 s activity with 50 ms bins [55]. (PDF) [file pcbi.1004759.s005.pdf]

**features**	**Spearman's ρ**	**P-value**
spike correlation and synaptic weight	$\rho = 0.32$	$P = 0.00$
spike correlation and separation	$\rho = -0.02$	$P = 0.04$
synaptic weight and separation	$\rho = -0.01$	$P = 0.46$
